# Ischemia Induces Release of Endogenous Amino Acids from the Cerebral Cortex and Cerebellum of Developing and Adult Mice

**DOI:** 10.1155/2013/839036

**Published:** 2013-01-10

**Authors:** Simo S. Oja, Pirjo Saransaari

**Affiliations:** ^1^Department of Paediatrics, Tampere University Hospital, 33521 Tampere, Finland; ^2^Medical School, University of Tampere, 33014 Tampere, Finland

## Abstract

Ischemia enhanced release of endogenous neuroactive amino acids from cerebellar and cerebral cortical slices. More glutamate was released in adult than developing mice. Taurine release enhanced by K^+^ stimulation and ischemia was more than one magnitude greater than that of GABA or glutamate in the developing cerebral cortex and cerebellum, while in adults the releases were almost comparable. Aspartate release was prominently enhanced by both ischemia and K^+^ stimulation in the adult cerebral cortex. In the cerebellum K^+^ stimulation and ischemia evoked almost 10-fold greater GABA release in 3-month olds than in 7-day olds. The release of taurine increased severalfold in the cerebellum of 7-day-old mice in high-K^+^ media, whereas the K^+^-evoked effect was rather small in adults. In 3-month-old mice no effects of K^+^ stimulation or ischemia were seen in the release of aspartate, glycine, glutamine, alanine, serine, or threonine. The releases from the cerebral cortex and cerebellum were markedly different and also differed between developing and adult mice. In developing mice only the release of inhibitory taurine may be large enough to counteract the harmful effects of excitatory amino acids in ischemia in both cerebral cortex and cerebellum, in particular since at that age the release of glutamate and aspartate cannot be described as massive.

## 1. Introduction 

Glutamate and *γ*-aminobutyrate (GABA) are the two major amino acid transmitters in the cerebral cortex and cerebellum, glutamate being responsible for excitatory and GABA for inhibitory transmission [1]. In these higher brain regions glycine was earlier assumed to be only an obligatory cotransmitter in the excitatory N-methyl-D-aspartate- (NMDA-) sensitive glutamate receptors, but more recent studies have also demonstrated the existence and function of strychnine-sensitive inhibitory glycine receptors in these structures [2, 3]. In addition to these established neurotransmitters, taurine also affects neuronal activity as an inhibitory modulator [4]. In the rodent brain the concentrations of taurine are high. In particular, in the developing brain it is the most abundant amino acid, even exceeding the concentration of glutamate [5]. 

The excessive extracellular accumulation of excitatory amino acids, predominantly that of glutamate but also of aspartate, in ischemia leads to cellular damage in the brain [6, 7]. Their massive release activates glutamate receptors, in particular those of the NMDA class [8], which leads to an excessive influx of Ca^2+^ and consequent adverse effects [9]. This excitotoxicity may be counteracted by the simultaneous enhanced release of inhibitory GABA and taurine [10, 11]. The functional status in the brain is a delicate balance between these excitatory and inhibitory neurotransmitters under both normal and pathological conditions.

In microdialysis studies *in vivo*, the overflow of endogenous extracellular amino acids can be assessed, (e.g., [6, 12]), but in the vast majority of *in vitro* studies the release of neurotransmitter amino acids has been investigated with the aid of preloaded radioactively labeled compounds. These admix more or less readily with the endogenous homologous pool in the cells and are only thereafter released into the extracellular spaces. Hence, the calculated release rate is affected by the efficacy of this mixing and the sizes of the endogenous amino acid pools and, thus, may not reliably reflect the magnitude of the release. The release of preloaded radioactively labeled amino acids from cerebral cortical preparations has been relatively frequently investigated, whereas relatively few studies have been made with the cerebellum [13–15]. Here we compared the release rates of endogenous GABA, glutamate, aspartate, glycine, and taurine from cerebral cortical and cerebellar slices to estimate the release on a molar basis under normoxic and ischemic conditions. The cerebral cortex and cerebellum represent functionally different brain structures, the cerebellum being predominantly inhibitory and the cerebral cortex with mixed functions, including excitation.

## 2. Experimental Procedure

### 2.1. Material

Developing (7-day olds) and young adult (3-month olds) NMRI mice of both sexes were used in the experiments. There were no significant differences between male and female mice. The results on male and female mice were therefore combined. The experiments conformed to the European Community Directive (86/609/EEC) for the ethical use of experimental animals, and they were approved by the Tampere University Committee for Animal Experiments. All efforts were made to reduce the number of animals used and their suffering.

### 2.2. Release Experiments

The mice were killed by fast decapitation and their brains excised without delay. Superficial slices 0.4 mm thick weighing 15–20 mg were manually prepared from the mouse cerebral cortex and cerebellum with a tissue slicer of Stadie-Riggs type. The slices were immediately immersed in 5 mL of preoxygenated medium and preincubated for 30 min under O_2_ at 37°C under agitation in standard medium containing (in mmol/L) NaCl 127, KCl 5, CaCl_2_ 0.8, MgSO_4_ 1.3, Na_2_HPO_4_ 1.3, N-2-hydroxyethylpiperazine-N′-2-ethanesulphonic acid (Hepes) 15, NaOH 11, and D-glucose 10 (pH 7.4). The slices were then transferred into 0.25 mL cups and superfused with the above medium (unless otherwise specified) at a rate of 0.25 mL/min for 50 min in a system in which freely floating shaken slices were kept under a continuous flow of oxygen in order to preserve their viability [16]. The superfusion medium was pooled during the first 20 min, whereafter 2 min fractions (0.5 mL) were collected. At 30 min the medium was in many experiments changed to medium containing 50 mM K^+^ (potassium stimulation). In our experimental set-up this K^+^ concentration has yielded the best and most reproducible responses in GABA and taurine release [16]. Under experimental conditions, designated as “ischemia,” the glucose-free medium was bubbled with N_2_ gas. The effluent fractions were subjected to amino acid assays by high-performance liquid chromatography. The amino acids eluted were visualized by means of o-phthaldialdehyde reagent and the results quantified with both external commercial standards and the internal standard of diamino-N-butyrate, as described in detail by Oja and Kontro [17]. The efflux of amino acids is either shown as a function of superfusion time (GABA, glutamate, and taurine) or calculated as an average efflux for the period of 32 to 50 min (glycine, glutamine, aspartate, alanine, serine, and threonine).

### 2.3. Statistical Calculations

The presence of statistically significant differences between the sample means was detected by the two-way analysis of variance. Comparison of individual means was made by Hartley's sequential method of testing.

## 3. Results

### 3.1. Cerebral Cortex

In 7-day-old mice taurine is the most prominent amino acid, followed by glutamate and aspartate (Table 1). The concentration of taurine decreases dramatically as the mice get older, when glutamate is the most abundant amino acid. The concentrations of glycine, aspartate, alanine, serine, and threonine are also significantly lower in 3-month-old mice, but no marked changes occurred in GABA, glutamate, and glutamine. It should be noted that the concentrations given in Table 1 represent the amino acid levels in the preincubated slices at the onset of superfusion, not the original tissue content. 

**Figure 2:**
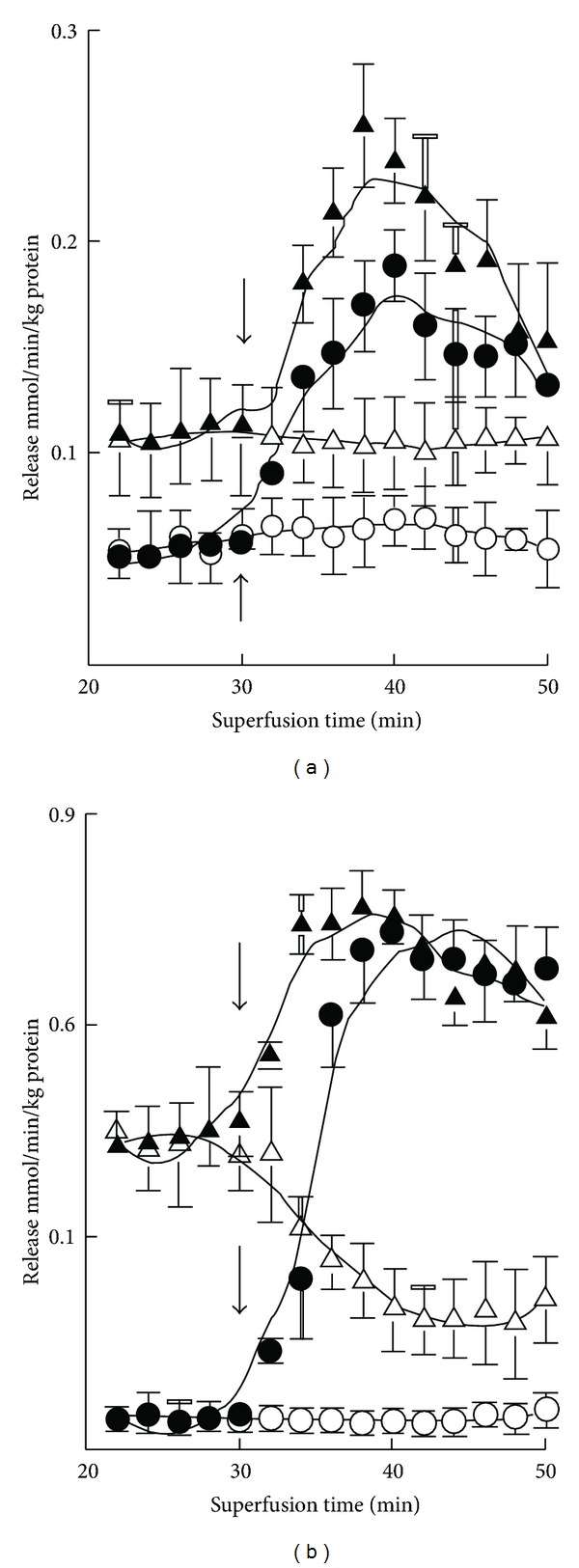
Time course of basal (-○-) and K^+^-evoked (-*⚫*-) GABA release from cerebral cortical slices in normoxia and basal (-∆-) and K^+^-stimulated (-▴-) GABA release in ischemia in 7-day-old (a) and 3-month-old (b) mice. The results are means of 4–8 independent experiments with SEMs indicated. Ischemia significantly (*P* < 0.01) enhanced the release at both ages. K^+^ stimulation was likewise significantly effective. Note the threefold difference in the scale of the *y*-axis in panels (a) and (b).

**Figure 3:**
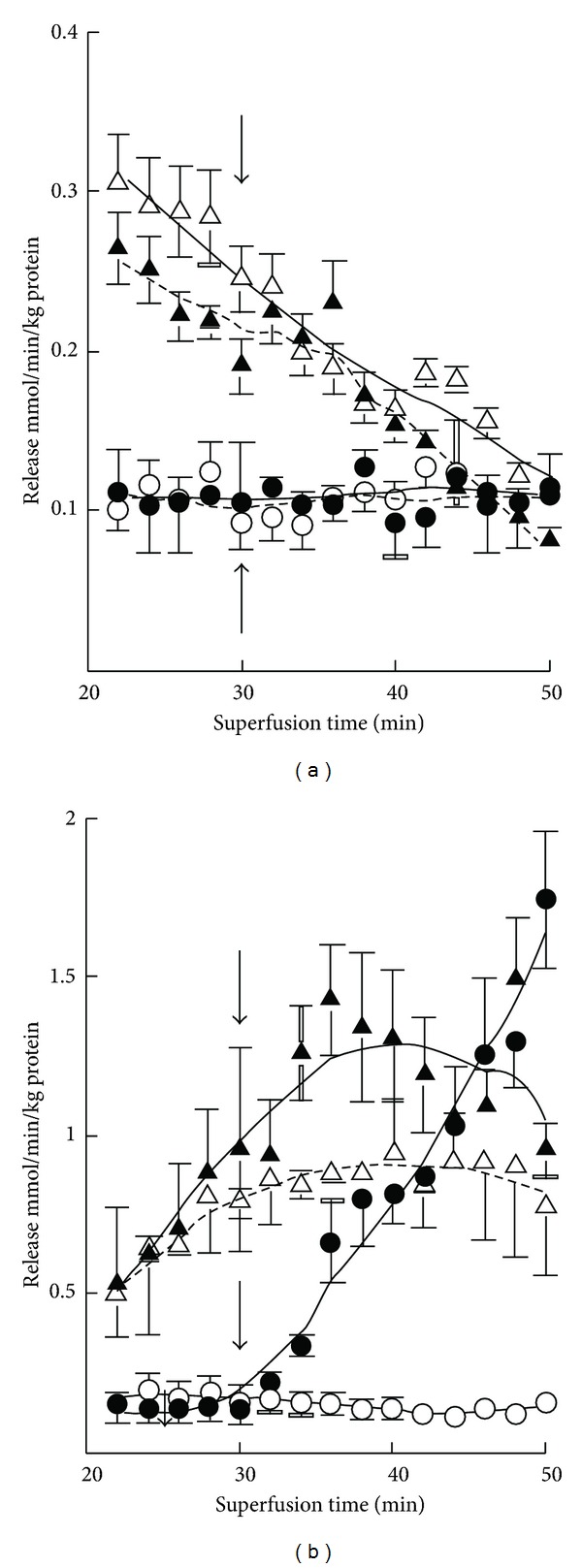
Time course of basal (-○-) and K^+^-evoked (-*⚫*-) glutamate release from cerebral cortical slices in normoxia and basal (-∆-) and K^+^-stimulated (-▴-) glutamate release in ischemia in 7-day-old (a) and 3-month-old (b) mice. The results are means of 4–8 independent experiments with SEMs indicated. Ischemia significantly (*P* < 0.01) enhanced the release in both age groups, but K^+^ stimulation was effective only in normoxia in both. Note the fivefold difference in the scale of the *y*-axis in panels (a) and (b).

**Figure 4:**
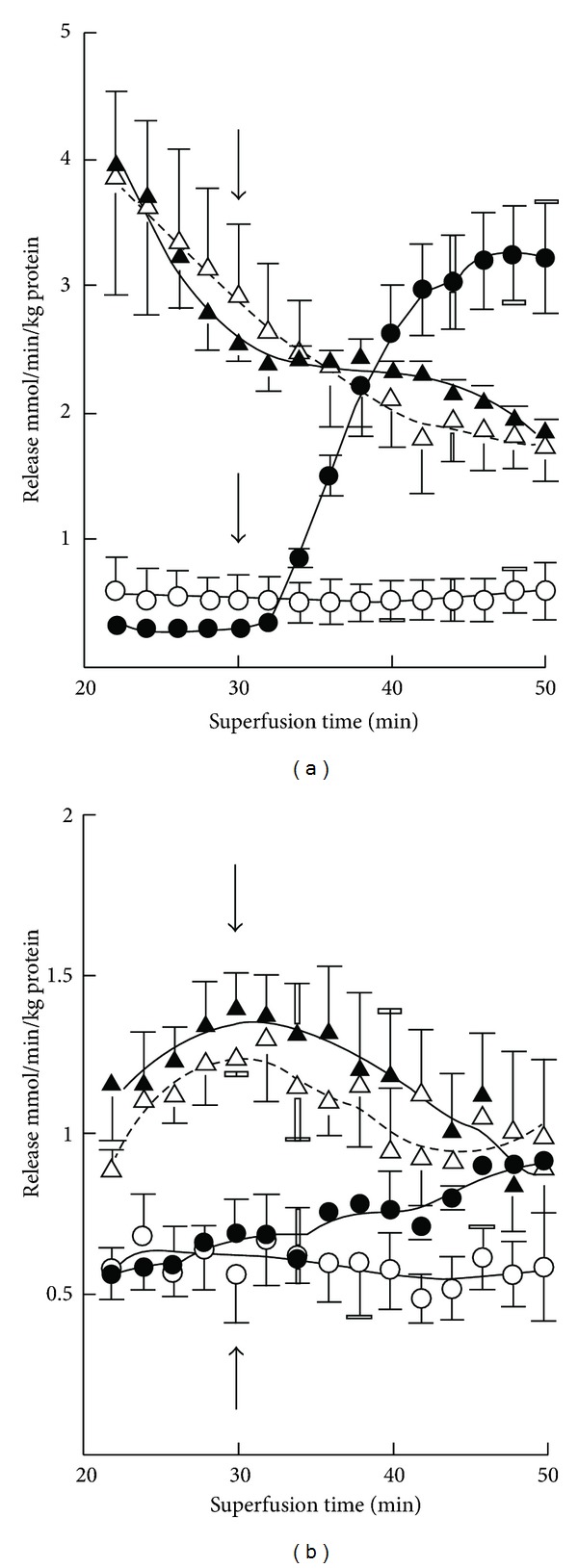
Time course of basal (-○-) and K^+^-evoked (-*⚫*-) taurine release from cerebral cortical slices in normoxia and basal (-∆-) and K^+^-stimulated (-▴-) taurine release in ischemia in 7-day-old (a) and 3-month-old (b) mice. The results are means of 4–8 independent experiments with SEMs indicated. Ischemia significantly (*P* < 0.01) enhanced the release in both age groups, but K^+^ stimulation was similarly effective only in normoxia, the effect being less pronounced (*P* < 0.05) in ischemia. Note the 2.5-fold difference in the scale of the *y*-axis in panels (a) and (b).

 The basal release of GABA was rather negligible in both 7-day- and 3-month-old mice, but K^+^ stimulation evoked marked release at both ages (Figure 2). The magnitude of the evoked release was however about fourfold greater in adult mice. Ischemia induced GABA release at both ages, more so in adults. K^+^ stimulation was clearly also preserved in ischemia in both age groups. In developing mice the release was stable during the experiments but in adults it was attenuated with prolonged ischemia (Table 2). In 7-day-old mice there occurred no enhancement of glutamate release upon K^+^ stimulation either from oxygenated or ischemic slices (Figure 3). However, the release was increased by ischemia at both ages. The increase was more pronounced during the early phase in developing mice (Table 2). In 3-month-old mice K^+^ stimulation was marked under normal incubation conditions. K^+^ stimulation was also effective in ischemia but the enhancement was less in magnitude. The basal release of taurine was markedly greater than that of GABA or glutamate (Figure 4). The release was increased severalfold when slices from 7-day-old mice were exposed to high-K^+^ medium, whereas the stimulation was rather small in 3-month olds. The release of taurine was markedly increased in ischemia in both developing and adult mice, but no K^+^-stimulated release was discernible at either age. In developing mice the release was somewhat diminished with prolonged ischemia (Table 2).

**Figure 5:**
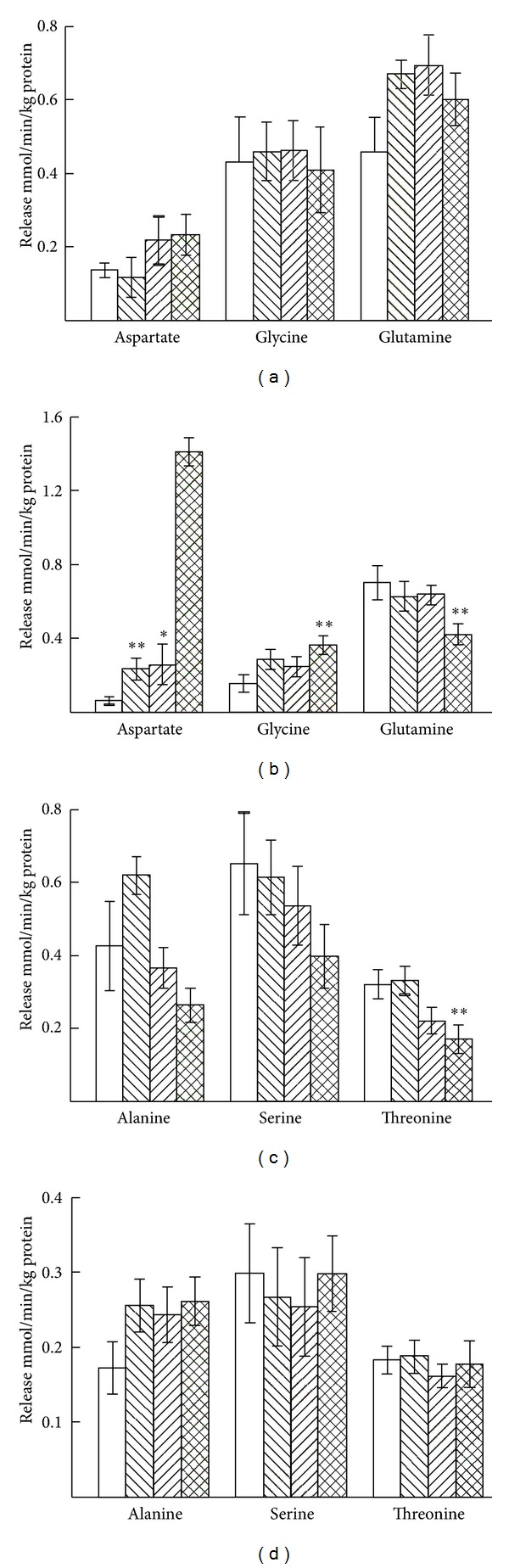
Average release of aspartate, glycine and glutamine (a) and alanine, serine and threonine (c) from cerebral cortical slices from 7-day-old mice and from cerebral cortical slices from 3-month-old mice ((b) and (d)) during the superfusion period of 32 to 50 min. The first open bars show the basal, and the second right-hatched bars the K^+^-stimulated release in normoxia, the third left-hatched bars the basal, and the fourth cross-hatched bars the K^+^-stimulated release in ischemia. The results are mean values ± SEM of 4–8 independent experiments. Note the twofold differences in the scale of the *y*-axes in panels (a), (b), (c), and (d). The statistically significant difference from the corresponding unstimulated release in normoxia: **P < 0.01.

In 7-day-old mice no effects of K^+^ stimulation or ischemia were seen in the release of aspartate, glycine, glutamine, alanine, and serine (Figure 5). On the other hand, aspartate release was enhanced by both treatments in 3-month-old mice, the combined effect of ischemia and K^+^ stimulation being particularly prominent. The release of glycine was also increased when slices from adult mice were exposed to K^+^ stimulation and ischemia, whereas the release of glutamine was diminished. No effects were seen in the release of alanine, serine, and threonine.

### 3.2. Cerebellum

Taurine is also the most prominent amino acid in the cerebellum of 7-day-old mice (Table 1). In 3-month olds glutamate is present at the highest concentration, followed by aspartate, glycine, and taurine. In the cerebellum the concentrations of GABA, glutamate, glutamine, and serine are at about the same level in both 7-day-old and 3-month-old mice, whereas the levels of taurine, glycine, aspartate, alanine, and threonine are lower in 3-month olds than in 7-day olds.

**Figure 1:**
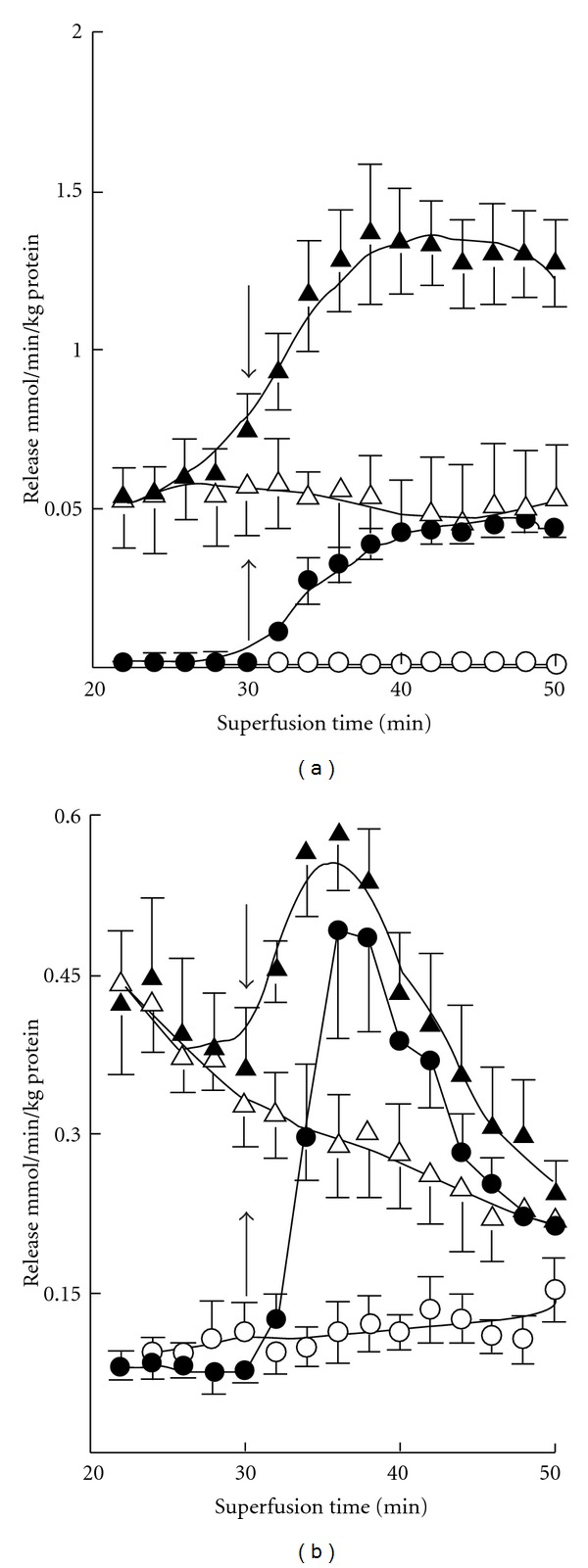
Time course of basal (-○-) and (-*⚫*-) K^+^-evoked GABA release from cerebellar slices in normoxia and basal (-∆-) and K^+^-stimulated (-▴-) GABA release in ischemia in 7-day-old (a) and 3-month-old (b) mice. The results are means of 4–8 independent experiments with SEMs indicated. Note the threefold differences in the scale of the *y*-axis in panels (a) and (b). Ischemia significantly (*P* < 0.01) enhanced the release at both ages. K^+^ stimulation was likewise significantly effective in both cases.

**Figure 6:**
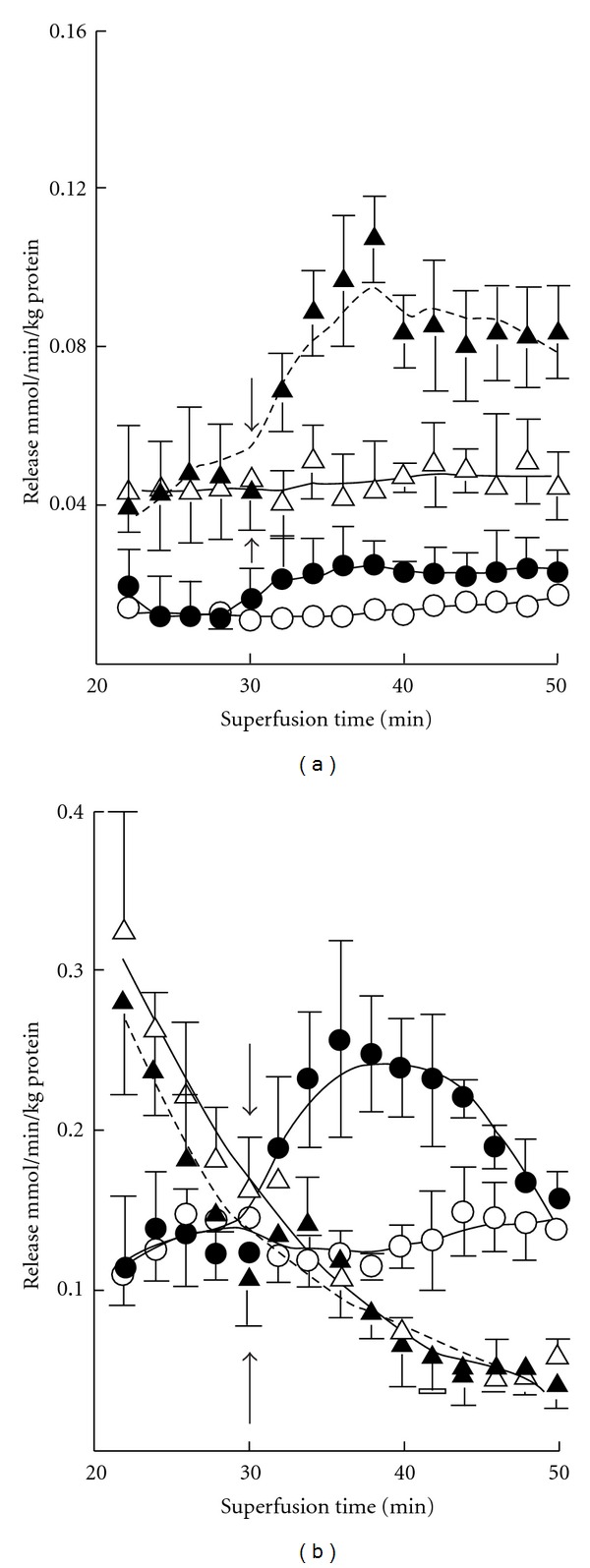
Time course of basal (-○-) and K^+^-evoked (-*⚫*-) glutamate release from cerebellar slices in normoxia and basal (-∆-) and K^+^-stimulated (-▴-) glycine release in ischemia in 7-day-old (a) and 3-month-old (b) mice. Note the fourfold differences in the scale of the *y*-axis in panels (a) and (b). The results are means of 4–8 independent experiments with SEMs indicated. Ischemia significantly (*P* < 0.01) enhanced the release in both age groups, but K^+^ stimulation was effective only in ischemia in 7-day-old mice and in normoxia in 3-month-old mice.

**Figure 7:**
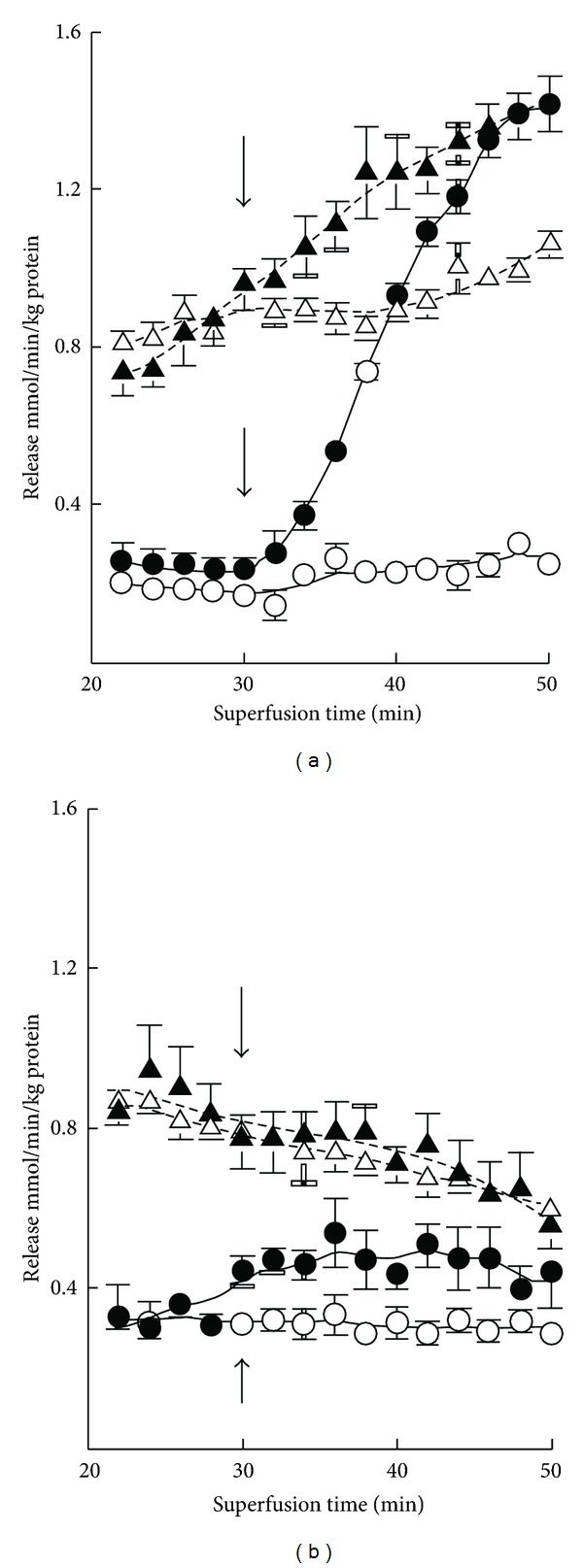
Time course of basal (-○-) and K^+^-evoked (-*⚫*-) taurine release from cerebellar slices in normoxia and basal (-∆-) and K^+^-stimulated (-▴-) taurine release in ischemia in 7-day-old (a) and 3-month-old (b) mice. The results are means of 4–8 independent experiments with SEMs indicated. Ischemia significantly (*P* < 0.01) enhanced the release in both age groups, but K^+^ stimulation had no significant effect in ischemia in 3-month-old mice.

There occurred only hardly detectable basal release of GABA from cerebellar slices from 7-day-old mice (Figure 1). K^+^ stimulation also evoked almost 10-fold greater GABA release in 3-month-old than in 7-day-old mice. Ischemia enhanced the release, and K^+^ stimulation was preserved in ischemic slices at both ages. However, the effects were much greater in adults in which the ischemia-induced enhancement was diminished with prolonged experiments (Table 2). The basal release of glutamate and the K^+^ stimulation were likewise severalfold greater in 3-month-old than in 7-day-old mice (Figure 6). The release of glutamate was enhanced in ischemia, but no K^+^-evoked release was discernible in adults. The ischemia-induced enhancement of release was greatly attenuated with time in adults (Table 2). The release of taurine increased several-fold in 7-day-old mice in high-K^+^ media, whereas K^+^ stimulation was rather small in magnitude in adults (Figure 7). The increase in release in ischemia was fairly stable during the experiments at both ages (Table 2). In ischemia K^+^ stimulation was partially preserved in 7-day-old mice, whereas no K^+^ effect was seen in 3-month-old mice (Figure 7).

**Figure 8:**
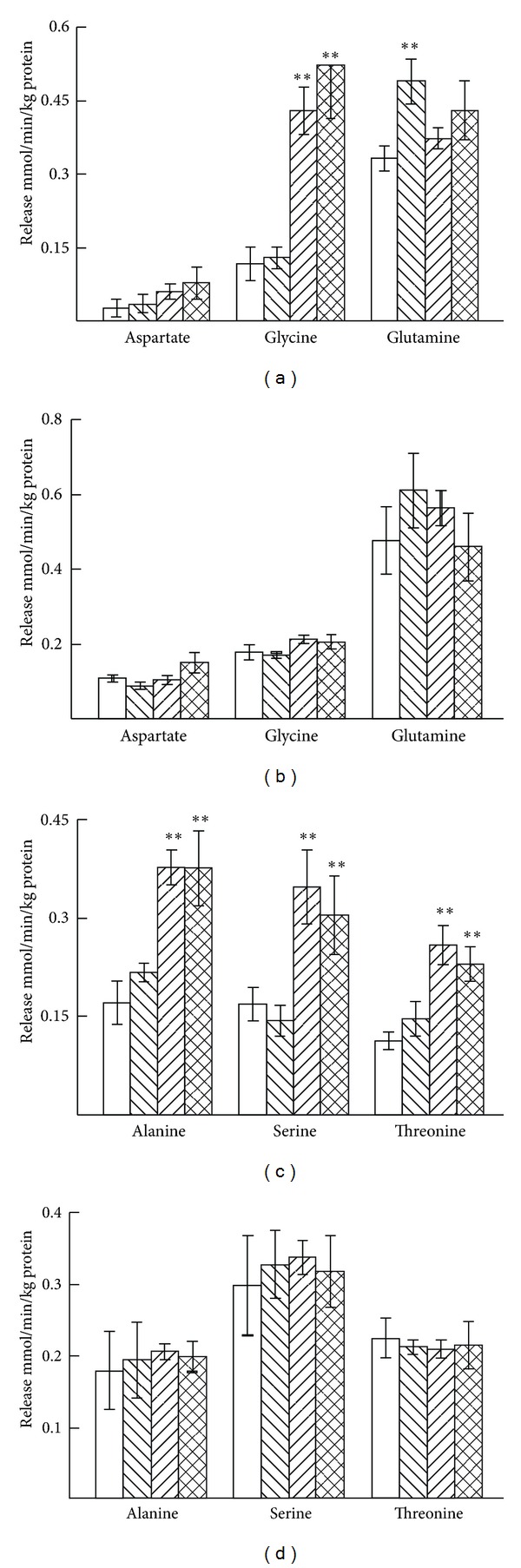
Average release of aspartate, glycine and glutamine (a) and alanine, serine and threonine (c) from cerebellar slices from 7-day-old mice and from cerebellar slices from 3-month-old mice ((b) and (d)) during the superfusion period of 32 to 50 min. The first open bars show the basal and the second right-hatched bars the K^+^-stimulated release in normoxia, the third left-hatched bars the basal, and the fourth cross-hatched bars the K^+^-stimulated release in ischemia. The results are mean values ± SEM of 4–8 independent experiments. The statistically significant difference from the corresponding unstimulated release in normoxia: *P < 0.05; **P < 0.01. Note the differences in the scale of the *y*-axes.

 Ischemia increased the release of glycine, glutamine, alanine, serine, and threonine in 7-day-old mice, but K^+^ stimulation had no effect on the release of these amino acids in ischemia (Figure 8). Aspartate release was in all cases somewhat marginal at this age. In 3-month-old mice no effects of K^+^ stimulation or ischemia were seen in the release of aspartate, glycine, glutamine, alanine, serine, or threonine.

## 4. Discussion

### 4.1. Mechanisms of Release

Although the amino acid levels in preincubated slices are not identical to the concentrations in intact tissue, the contents measured here reflect those obtaining in vivo. A characteristic finding is the very high taurine concentration in the developing mouse brain. In spite of the much higher content in the developing cerebral cortex and cerebellum, the basal release rates of endogenous taurine were not markedly different, but the high content to a great extent obviously underlies the several-fold greater release evoked by K^+^ stimulation and ischemia. These effects were clearly more marked in the cerebral cortex than in the cerebellum in both experimental groups. However, the differences in concentration of other amino acids between developing and adult mice are also in any case of such magnitude that they will have affected the present estimated release rates.

Ischemia induced the release of neuroactive amino acids in all brain areas studied. This release could result, for instance, from destabilization and deterioration of plasma membranes owing to phospholipid hydrolysis by phospholipases, resulting in diffusion of amino acids along their concentration gradients [18]. However, in ischemia here there occurred no measurable increase in the extrusion of lactate dehydrogenase (a common marker of plasma membrane damage and nonspecific lysis of neural cells) from the slices upon incubation (data not shown). Nor has it been increased in other experiments of the present type [19, 20]. However, longer periods of ischemia are associated with increasing degrees of plasma membrane disruption, allowing for a greater leakage of intracellular contents [21]. In keeping with the assumption that deterioration of cell plasma membranes may not have been a major factor underlying amino acid release, there now occurred no increase in the release of the nontransmitter amino acids, alanine, serine, and threonine in the cerebral cortex in ischemia in either experimental group. However, the release of all these amino acids increased in ischemia in slices prepared from the developing cerebellum. It is thus reasonable to surmise that any major damage to neural membranes will not have been involved in the present release rates of amino acids, with a reservation to the preparations from the developing cerebellum.

The opening of swelling-induced Cl^−^ channels also allows the passage of amino acids down their concentration gradients. Edema is a frequent consequence of cerebral ischemia [22]. When cells swell, they attempt to restore their normal volume by extruding osmotically active solutes such as amino acids [23]. In particular, release of taurine has often been shown to be evoked by cell swelling in the cerebral cortex, exhibiting typically a delayed time course [24, 25], similar to those obtained in the present study. Moreover, the apparent release of amino acids from slices also depends on the efficacy of reuptake. The amino acids released must traverse the extracellular spaces to reach the medium and be detected. This is the reason why uptake mechanisms also affect the estimated release rates. 

In addition to the cell plasma membrane damage discussed previously, and in the case of neurotransmitter amino acids Ca^2+^-dependent exocytosis from synaptic vesicles [26], the mechanisms involved in the release of amino acids in ischemia may include swelling-evoked release via anion channels [27], reversal of amino acid transporters in depolarized cells [28], or acidosis-induced failure of reuptake into neurons and glia [29]. In particular, the release of glutamate and aspartate has been postulated to involve, in addition to Ca^2+^-dependent exocytotic release, Ca^2+^-independent release due to a depolarization-induced reversal of the Na^+^-dependent high affinity acidic amino acid transporters [30, 31]. Ca^2+^-dependent release may account for the initial efflux of neurotransmitter amino acids at the onset of ischemia, whereas Ca^2+^-independent nonvesicular release, mediated by Na^+^-dependent amino acid transporters in plasma membranes operating in a reversed mode, could be mostly responsible for the later phase of release [32]. This may signify that the release of glutamate from cerebellar slices could have been due mainly to exocytosis in the present experiments, whereas in cerebral cortical slices the function of transporters may have markedly contributed. 

### 4.2. Ischemia and Excitotoxicity

Excessive release of the excitotoxic amino acids glutamate and aspartate is considered to be a significant contributor to neuronal death in the ischemic brain [33, 34]. There is evidence for a positive feedback loop, in which glutamatergic neurons can be stimulated to release additional glutamate as a consequence of activation of its receptors [35, 36]. In particular, the N-methyl-D-aspartate (NMDA) receptors may occupy a central position here, since they have been shown to regulate the release of both excitatory and inhibitory amino acids from rat fetal cortical neurons [37]. In human cerebral cortical slices K^+^ stimulation and ischemia have also markedly enhanced the release of glutamate, aspartate, GABA, and glycine, but not that of glutamine. The K^+^-evoked release was Ca^2+^-dependent, whereas the ischemia-induced release was not [38]. Substantial amounts of glutamate were now released in the adult cerebral cortex under ischemic conditions, and the K^+^ stimulation was partially preserved, but the release could not be described as massive in comparison with the release of GABA or taurine. The release of glutamate was markedly smaller in the developing cerebral cortex. In the cerebellum the release was likewise greater in adults, being however significantly smaller than in the cerebral cortex and rapidly attenuated with time. This finding may indicate that the adult cerebral cortex is more susceptible to excitotoxicity. The marked release of aspartate, in particular with the concomitant K^+^ stimulation, could contribute to this effect in the cerebral cortex, but not in the cerebellum. 

The release of GABA from cerebral cortical slices was greater that that from cerebellar slices in both experimental groups. In keeping with this finding, glutamate decarboxylase-positive cell bodies, that is, GABAergic neuronal density, in the rat neocortex are high already at birth [39]. On the other hand, in adult mice GABA release was of the same order of magnitude in both brain areas. In adults, the release of glutamate was significantly greater than that of GABA. In the adult cortex both these neurotransmitters function in the vast majority of all synapses, but the glutamatergic synapses outnumber the GABA synapses about fivefold [40]. The greater release of glutamate thus reflects this relation. In cultured rat cerebellar neurons the release of GABA and taurine has been shown to originate from the GABAergic interneurons, the basket and stellate cells, and the release of glutamate and aspartate mainly from the granule neurons [41]. In cultured neurons from the rat cerebellum the glutamate receptor agonists kainate and quisqualate have evoked the release of endogenous glutamate, taurine, and GABA in a dose- and Ca^2+^-dependent manner [42, 43]. Endogenous aspartate, glutamate, and GABA are also released from such cultures by K^+^ depolarization in a dose- and Ca^2+^-dependent manner whereas the release of taurine is dose-dependent but not Ca^2+^-dependent [44]. In contrast, the omission of Ca^2+^ has enhanced basal taurine release, a finding also recorded, for example, in cerebral cortical slices and in hippocampal slices prepared from young, adult, and aged mice [45, 46].

The greater glycine release from cortical slices from developing than from adult mice may be associated, in addition to the high glycine content, with the presence of glycine receptors in the cerebral cortex during early development [47, 48]. In particular, the *α*2 subunit of glycine receptors declines sharply following the first postnatal week and remains at a low level in the adult neocortex [47, 49, 50]. Moreover, the glycine sensitivity of neurons in the rat prefrontal cortex has been shown to decrease with age [51], and functional glycine receptors in older animals have not been demonstrated. On the other hand, the expression of glycine receptors in the cerebellum is rather weak and does not show a similar decline with maturation [52]. 

Taurine acts at both GABA and glycine receptors, being however less effective than either of these [3, 53]. It therefore acts as an inhibitory agent in the central nervous system [4]. The release of taurine in the developing cerebral cortex and cerebellum in ischemia is of such magnitude that it could clearly counteract the effects of excitatory amino acid transmitters, in particular since at that age the release of glutamate and aspartate cannot be described to be massive. GABA and glycine cannot protect against excitotoxicity in the developing cerebral cortex and cerebellum, since GABA is during early development rather excitatory than inhibitory [54, 55]. Glycine receptors also tend to mediate excitation in the neonatal rat cerebral cortex [56, 57].

In conclusion, we may state that the releases from the cerebral cortex and cerebellum were markedly different and also differed between developing and adult mice. In developing mice with 7 days of age, only the release of inhibitory taurine may be large enough to counteract the harmful effects of excitatory amino acids in ischemia in both cerebral cortex and cerebellum, in particular since at that age the release of glutamate and aspartate cannot be described as massive. The present experiments in which rather long exposure times were studied cannot reveal any fast or transient effects on amino acid release. Moreover, it is open to what extent the results obtained are applicable to the human brain, though the basic mechanisms are likely similar in the mouse and the man.

## Figures and Tables

**Table 1:** Amino acid contents in slices.

Amino acid (mmol/kg)	Cerebral cortex		Cerebellum	
	7-day olds	3-month olds	7-day olds	3-month olds
Taurine	296.19 ± 23.41	26.81 ± 2.75*	194.37 ± 12.71	13.17 ± 3.06*
GABA	16.38 ± 1.13	18.41 ± 1.36	13.25 ± 1.80	16.03 ± 1.88
Glycine	24.17 ± 7.42	3.82 ± 0.27*	18.23 ± 1.49	8.52 ± 1.08*
Aspartate	57.82 ± 4.25	20.92 ± 1.54*	50.26 ± 2.64	17.78 ± 1.90*
Glutamate	75.13 ± 5.71	64.99 ± 3.38	54.82 ± 3.18	53.70 ± 6.40
Glutamine	7.54 ± 0.68	6.53 ± 1.03	9.58 ± 1.60	6.26 ± 1.27
Alanine	10.34 ± 3.38	3.62 ± 0.29*	8.50 ± 0.38	4.99 ± 0.74*
Serine	21.33 ± 9.59	5.58 ± 0.40*	9.93 ± 0.38	9.55 ± 1.62
Threonine	5.93 ± 2.06	2.13 ± 0.16*	5.31 ± 0.20	3.11 ± 0.84*

Amino acid concentrations (mean values ± SEM) are given in mmol/kg protein in preincubated slices; that is, they show the amino acid contents before the onset of superfusion. Number of independent experiments 8 (cerebral cortex) and 16 (cerebellum). Significance of differences between 7-day-old and 3-month-old mice: *P < 0.01.

**Table 2:** Early and late ischemia-induced release of GABA, glutamate, and taurine.

	7-day-old mice		3-month-old mice	
Amino acid	mmol/min/kg protein		mmol/min/kg protein	
	Release period		Release period	
	20–30 min	32–50 min	20–30 min	32–50 min
Cerebral cortex				
GABA	0.108 ± 0.002	0.104 ± 0.001	0.429 ± 0.007	0.235 ± 0.024*
Glutamate	0.282 ± 0.010	0.171 ± 0.012*	0.671 ± 0.057	0.870 ± 0.015*
Taurine	3.357 ± 0.164	2.078 ± 0.100*	1.112 ± 0.061	1.050 ± 0.038
Cerebellum				
GABA	0.055 ± 0.001	0.051 ± 0.001	0.384 ± 0.020	0.265 ± 0.011*
Glutamate	0.044 ± 0.001	0.046 ± 0.001	0.229 ± 0.029	0.082 ± 0.014*
Taurine	0.859 ± 0.028	0.933 ± 0.022	0.826 ± 0.015	0.687 ± 0.018*

The average release rates (±SEM) were calculated for the release periods indicated. Number of independent experiments 8 and release estimates 5 (20–30 min) and 10 (32–50 min). Significance of differences between the early and late release periods: *P < 0.01.
